# ITGA2 as a potential nanotherapeutic target for glioblastoma

**DOI:** 10.1038/s41598-019-42643-7

**Published:** 2019-04-17

**Authors:** Peng Guo, Alexander Moses-Gardner, Jing Huang, Edward R. Smith, Marsha A. Moses

**Affiliations:** 1 Vascular Biology Program, Boston Children’s Hospital, 300 Longwood Avenue, Boston, MA 02115 United States; 2 Department of Surgery, Harvard Medical School and Boston Children’s Hospital, 300 Longwood Avenue, Boston, MA 02115 United States; 3 Department of Neurosurgery, Harvard Medical School and Boston Children’s Hospital, 300 Longwood Avenue, Boston, MA 02115 United States

**Keywords:** CNS cancer, Drug delivery

## Abstract

High grade gliomas, including glioblastoma (GBM), are the most common and deadly brain cancers in adults. Here, we performed a quantitative and unbiased screening of 70 cancer-related antigens using comparative flow cytometry and, for the first time, identified integrin alpha-2 (ITGA2) as a novel molecular target for GBM. In comparison to epidermal growth factor receptor (EGFR), a well-established GBM target, ITGA2 is significantly more expressed on human GBM cells and significantly less expressed on normal human glial cells. We also found that ITGA2 antibody blockade significantly impedes GBM cell migration but not GBM cell proliferation. To investigate the utility of ITGA2 as a therapeutic target in GBM, we designed and engineered an ITGA2 antibody-directed liposome that can selectively deliver doxorubicin, a standard-of-care chemotherapeutic agent, to GBM cells. This novel approach significantly improved antitumor efficacy. We also demonstrated that these ITGA2 antibody-directed liposomes can effectively breach the blood-brain tumor barrier (BBTB) *in vitro* via GBM-induced angiogenesis effects. These findings support further research into the use of ITGA2 as a novel nanotherapeutic target for GBM.

## Introduction

High grade gliomas, including glioblastoma (GBM), are the most common brain cancers in adults, representing between 15 and 20% of all brain tumors diagnosed, including a significant fraction of pediatric cases^1–4^. It is also among the deadliest tumor types, with only approximately 5% of diagnosed GBM patients surviving 5 years post-diagnosis^3,4^. Treatment for this type of tumor involves brain surgery, typically paired with chemotherapy and radiation, which can be associated with severe adverse effects and provides only limited therapeutic efficacy. To date, there is still no effective targeted therapeutic to treat GBM in the clinic, highlighting urgent and significant needs to identify new GBM targets and to develop novel GBM-targeted therapeutics.

Cancer nanomedicine is a revolutionary approach that has emerged in the past two decades and is changing the paradigm of cancer treatment^5–7^. The recent rapid development of nanomaterials has created a promising opportunity to engineer “virus-like” nanovehicles (termed nanomedicines) to circulate in the body and selectively deliver various therapeutic, diagnostic, and theranostic agents to the diseased sites (e.g., tumor and metastatic lesions) while sparing healthy organs and tissues. First-generation nanomedicines such as Doxil, a PEGylated liposomal doxorubicin, have been approved by the United States Food and Drug Administration (USFDA) and the European Medical Agency (EMA) for treating ovarian and breast cancers^8^, largely because these drugs exhibit fewer adverse effects and better safety profiles than conventional chemotherapy regimens. However, Doxil failed to produce significant improvements in clinical outcomes for GBM patients in Phase II clinical trials^9–11^. Similar results were also observed for a non-targeting liposomal temozolomide in a recent pre-clinical GBM study^12^. We reasoned that these unsuccessful results might be due to the fact that these nanomedicines deliver their payloads in a non-specific, non-targeted manner and that drug availability to the GBM may be severely hindered by blood-brain tumor barrier (BBTB) and tumor heterogeneity. To resolve these issues, we hypothesized that functionalizing non-specific nanomedicines with antibodies against GBM-specific antigens can guide them to selectively recognize and ablate GBM tumors in a more precise and efficient manner.

In this study, we identified the cell surface antigen ITGA2 as a novel molecular target highly specific for GBM and conserved across multiple cell lines and patient samples. We have investigated the effects of ITGA2 blockade on inhibiting tumor cell function and have demonstrated a clinical correlation between ITGA2 expression and patient prognosis. Finally, we reported our experience in combining ITGA2-specific targeting with an engineered liposomal nanomedicine capable of crossing the BBTB *in vitro* and effectively target GBM cells.

## Results

### Identification of ITGA2 as a novel GBM target

**Figure 1:**
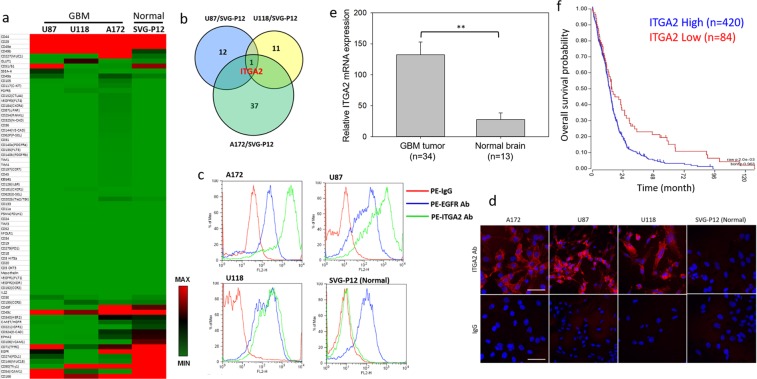
Identification of ITGA2 as a molecular target for GBM. (**a**) Surface protein expression profile of 70 cancer-related antigens in three human GBM cell lines and normal SVG-P12 cells. (**b**) Venn diagram indicating ITGA2 co-overexpressed by three GBM cell lines in reference to SVG-P12 cells. (**c**) Cell membrane expression levels of ITGA2 and EGFR in GBM and SVG-P12 cells using flow cytometry analysis, showing increased tumor-specificity of ITGA2 expression on GBM cells as compared to both control cells and also to the current GBM marker, EGFR. (**d**) Representative microscopic images of immunofluorescent staining of ITGA2 in GBM and SVG-P12 cells. Scale bars represent 50 µm. (**e**) ITGA2 mRNA expression levels in human GBM tumor tissues and normal brain tissues. **P < 0.001. (**f**) Correlation between overall survival and ITGA2 mRNA expression levels in GBM patients as determined via Kaplan-Meier analysis (Log-rank test). Data of (**e**) and (**f**) were obtained from the R2: Genomics Analysis and Visualization Platform database.

Identifying new GBM targets holds the key to the development of GBM-targeted therapeutics. Thus, we performed an unbiased and quantitative screening of a panel of 70 cancer-related cell surface antigens on three well-established human GBM cell lines (A172, U87, and U118) in comparison with non-neoplastic human glial SVG-P12 cells (normal control) by flow cytometric analysis as previously reported^13^ (Fig. 1a). Of the 70 screened targets, ITGA2 was identified as the only target that commonly overexpressed in all three GBM cell lines (Fig. 1b) and was selected for further investigation. Given that EGFR has been known as a well-established target for GBM^14–16^, we compared the overexpression levels of ITGA2 and EGFR in the three GBM cell lines and healthy SVG-P12 cells. The overexpression of ITGA2 was found to significantly exceed that of EGFR’s (Fig. 1c), on two GBM cell lines (A172 and U87) and to be at an equivalent level on U118 cells. It is noteworthy that EGFR is highly overexpressed on healthy SVG-P12 cells which may cause off-target toxicity for EGFR-targeted therapy, whereas there is no ITGA2 expression on SVG-P12 cells. These results suggest that ITGA2 may be a novel GBM-specific target. Furthermore, we performed immunofluorescent staining of ITGA2 on GBM cells. As observed in Fig. 1d, ITGA2 expression is localized on the plasma membranes of the three GBM cell lines (A172, U87, and U118) but absent on SVG-P12 cells, which is readily accessible to ITGA2 antibody-directed nanomedicines. To correlate our findings with GBM clinic data, we compared ITGA2 mRNA expression levels in human GBM tumors and normal brain tissues by querying the R2: Genomics Analysis and Visualization Platform database (https://hgserver1.amc.nl/, Datasheet: Mixed Pediatric Brain (Normal-Tumor)-Donson-130-MAS5.0-u133p2)^17,18^. As demonstrated in Fig. 1e, we confirmed that ITGA2 expression is significantly upregulated in human GBM tumors (n = 34) in comparison with normal brain tissues (n = 13), which is consistent with our *in vitro* findings. Furthermore, we analyzed the potential impact of ITGA2 on the overall survival of GBM patients using the same database (a cohort of 540 patients, Datasheet: Tumor Glioblastoma-TCGA-540-MAS5.0-u133a). As shown in Fig. 1f, GBM patients (n = 420) with high ITGA2 expression demonstrated a significantly worse prognosis than the low ITGA2 group (n = 84, P = 0.002, log-rank test), indicating that ITGA2 may also serve as an important clinical biomarker of poor prognosis in GBM patients.

### Therapeutic functions of ITGA2 blockade

**Figure 2:**
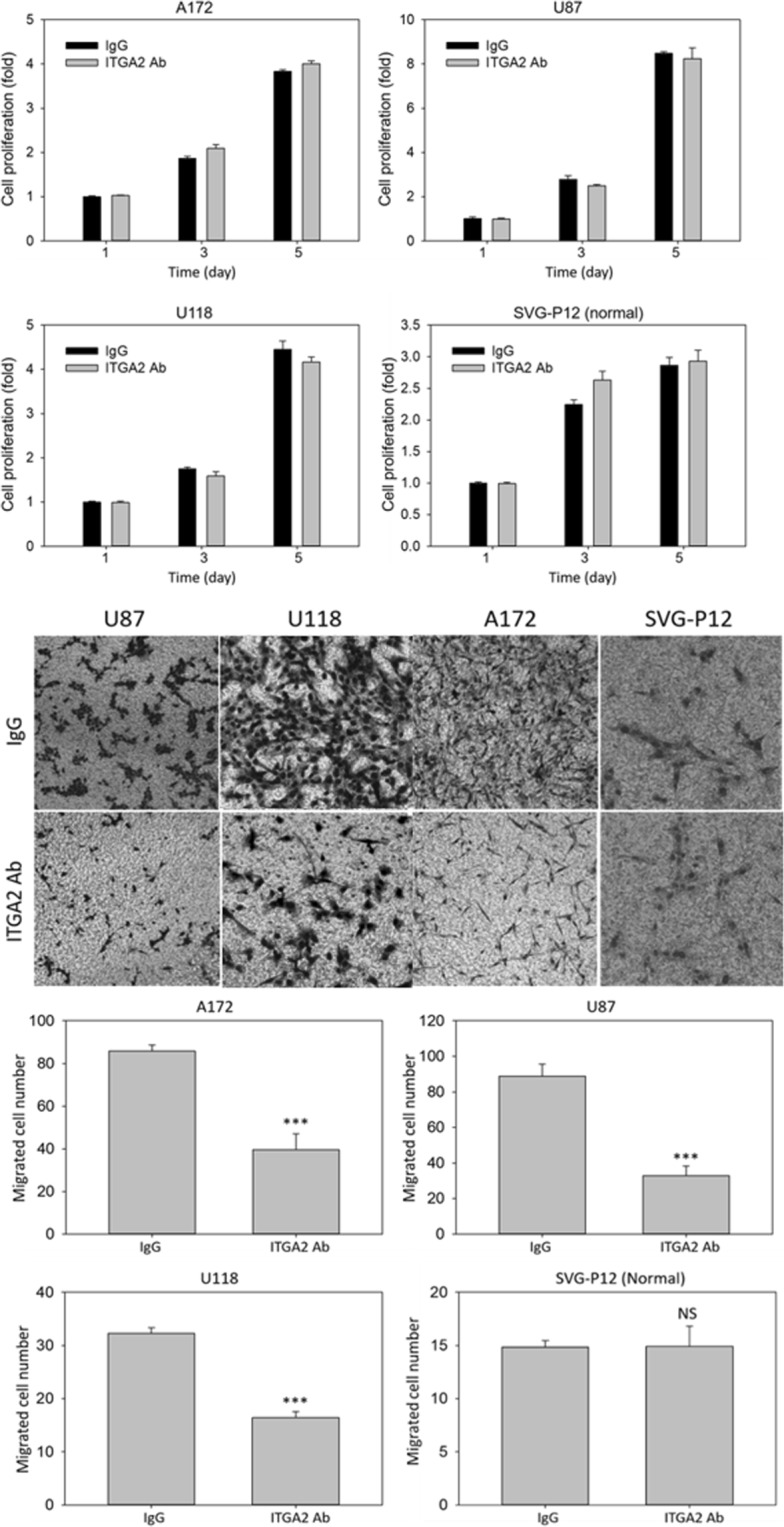
Therapeutic functions of ITGA2 blockade. (**a**) The effect of ITGA2 antibody on GBM cell proliferation. Representative microscope images (**b**) and quantitative analyses (**c**) of migrated GBM cells under ITGA2 antibody blockade treatment in a transwell migration assay. Significant reductions in GBM migration were observed with ITGA2 blockade. IgG was used as a control. ***P < 0.001, NS not significant.

We evaluated the therapeutic potential of antibody blockade of ITGA2 on two key malignant characteristics of GBM cells: proliferation and migration. As presented in Fig. 2a, we found that the treatment of ITGA2 antibody (2 µg/mL) does not induce any significant alternation in GBM (A172, U87 and U118) and normal glial SVG-P12 cell proliferation. However, as shown in Fig. 2b, ITGA2 antibody (2 µg/mL) significantly impeded the GBM cell migration in comparison with non-specific IgG. We found that approximately 50–60% of GBM cell migration was inhibited by ITGA2 antibody blockade whereas no significant effect was observed in normal SVG-P12 cells (Fig. 2c). We further evaluated different time points (day 1, day 3, and day 5) and the effects of varying antibody concentrations (0.4, 2, and 10 µg/mL) on human GBM (A172) cell proliferation (Fig. S1a–c). Similarly, we found that administration of ITGA2 antibody does not affect cell proliferation when compared to with control IgG treatment at all tested time points and antibody concentrations. However, when we evaluated the effects of antibody concentration on A172 cell migration, we observed that the inhibitory effect of ITGA2 antibody on GBM cell migration positively correlates with its antibody concentration (Fig. S2a,b). We reasoned that using antibodies to block ITGA2 may abrogate GBM cell migration and subsequently a pro-metastatic phenotype. Similar effects were also observed in ITGA2 antibody blocking studies of gastric cancer cells^19^. ITGA2 antibody showed no inhibitory effect on SVG-P12 cells due to its absence of ITGA2 expression. These studies suggested that ITGA2 antibody not only serves as a nanomedicine binding ligand for GBM recognition, but that it may also hinder disease progression.

### Development of ITGA2 antibody-directed liposomes

**Figure 3:**
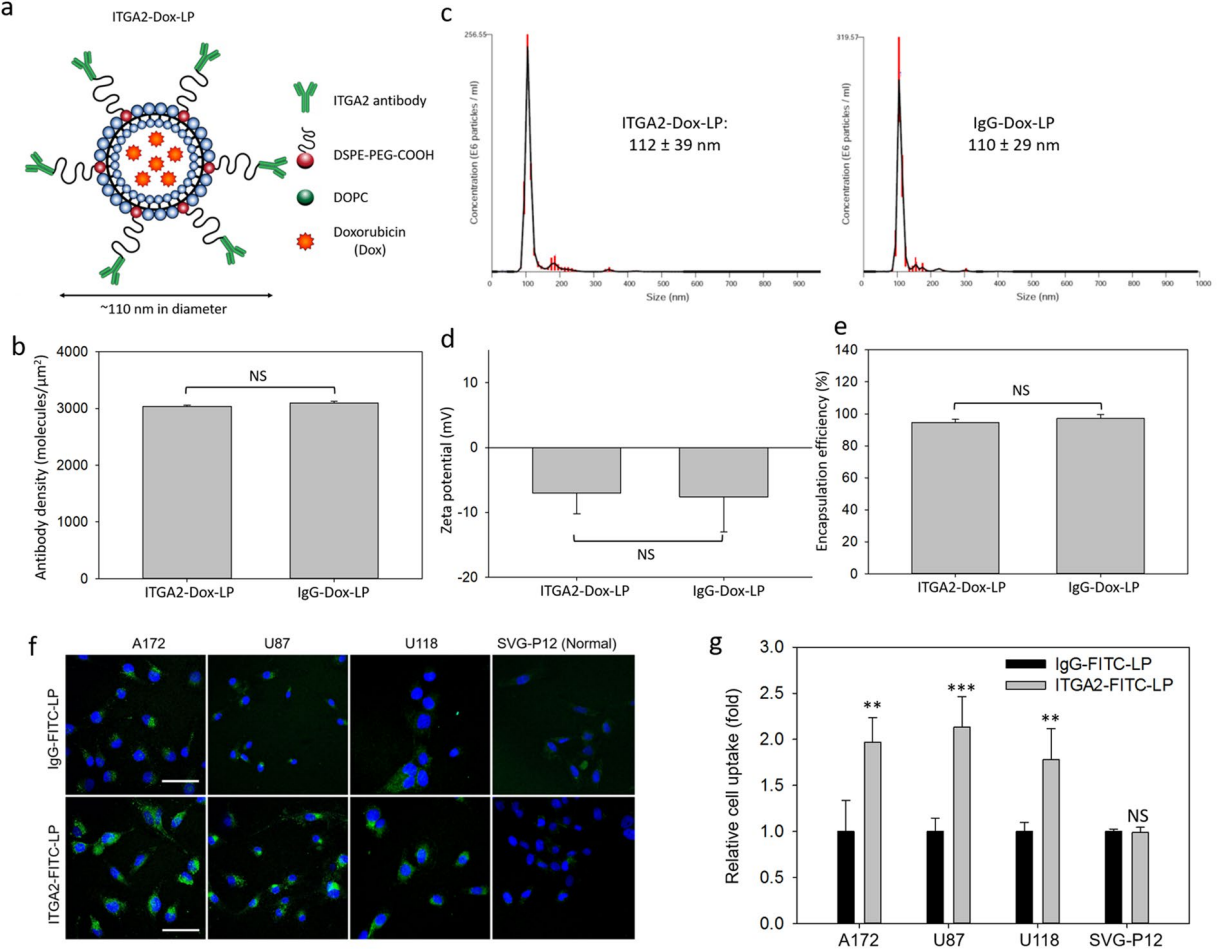
Development of an ITGA2 antibody-directed nanomedicine. (**a**) Schematic illustration of ITGA2 antibody-conjugated, doxorubicin-encapsulating liposomes (ITGA2-Dox-LP). (**b**) The surface density of ITGA2 antibody or IgG-conjugated on ITGA2-Dox-LP or IgG-Dox-LP quantified using a microbead assay. Hydrodynamic diameter (**c**) and zeta-potential (**d**) of ITGA2-Dox-LP and IgG-Dox-LP quantified using dynamic light scattering measurement. (**e**) The encapsulating efficiency of doxorubicin in ITGA2-Dox-LP and IgG-Dox-LP. (**f**) Representative fluorescent images of GBM and SVG-P12 cells uptaking fluorescent-labeled ITGA2-FITC-LP and IgG-FITC-LP. The scale bars represent 50 µm. (**g**) Quantitative GBM cell uptake of ITGA2-FITC-LP in comparison to non-specific IgG-FITC-LP. NS, not significant; **P < 0.01; ***P < 0.001.

To translate the use of ITGA2 into GBM-targeted therapy, we developed an ITGA2 antibody-directed liposome (ITGA2-Dox-LP) to selectively deliver doxorubicin, a standard-of-care chemodrug, to GBM cells. ITGA-Dox-LP was engineered using a nanoporous membrane extrusion method as we previously reported^20–24^; non-specific IgG conjugated, doxorubicin-encapsulating liposomes (IgG-Dox-LPs) were also prepared and tested as controls. As shown in Fig. 3a, ITGA-Dox-LPs were comprised of 95 mol% of 1,2-dioleoyl-sn-glycero-3-phosphocholine (DOPC) and 5 mol% of 1,2-distearoyl-sn-glycero-3-phosphoethanolamine-N-carboxy(polyethylene glycol) (DSPE-PEG-COOH). The hydrophilic PEG chain (2 kDa) in DSPE-PEG-COOH helps to extend its blood circulation time^25^. ITGA2 antibodies were covalently conjugated with the carboxylate group of DSPE-PEG-COOH via EDC/NHS chemistry. The resulting ITGA2 antibody density on the liposome surface was determined to be approximately 3000 antibodies per µm^2^, equivalent to 114 antibodies per liposomes (Fig. 3b). The average hydrodynamic diameters of ITGA2-Dox-LP and IgG-Dox-LP are highly similar at approximately 110 nm with a narrow size distribution (Fig. 3c). Both ITGA2-Dox-LP and IgG-Dox-LP are slightly negative charged with a similar zeta potential of approximately −7 mV (Fig. 3d). Doxorubicin was actively loaded into liposomes using a transmembrane gradient assay^26^, resulting in an extremely high encapsulating efficiency of over 95% in both prepared liposomes (Fig. 3e). We further characterized the morphology and structure of ITGA2-Dox-LP and IgG-Dox-LP using transmission electron microscopy (TEM). Lipid bilayers and encapsulated doxorubicin nanoprecipitates of liposomes can be visualized in Fig. S3.

### GBM specificity of ITGA2 antibody-directed liposomes

We determined the GBM specificity of ITGA2-Dox-LP by comparing its cell uptake in both GBM and healthy glial cells. In order to avoid cytotoxicity-mediated interference of the chemotherapeutic agent on cell uptake, we used FITC-dextran, a non-cytotoxic fluorescent dye, to replace doxorubicin in the liposome preparation. The obtained FITC-dextran encapsulating, ITGA2 antibody or IgG-conjugated liposomes (ITGA2-FITC-LP or IgG-FITC-LP) were incubated with three GBM cell lines (A172, U87, and U118) and SVG-P12 cells, and we quantified the cell uptake of these liposomes by measuring the cellular fluorescent intensity changes. The immunofluorescent staining images in Fig. 3f showed that three GBM cell lines (A172, U87, and U118) internalized substantially more ITGA2-FITC-LPs than non-specific IgG-FITC-LP, whereas normal SVG-P12 cells exhibited no preferable uptake of ITGA2-FITC-LP due their lack expression of ITGA2. The relative cell uptake was quantified and showed in Fig. 3g, and ITGA2-FITC-LP showed approximately 75% to 150% increases in GBM cell uptake than non-specific IgG-FITC-LP. These results indicate that ITGA2 antibody-directed liposomes can selectively bind and deliver therapeutic agents to GBM cells.

### GBM-specific toxicity of ITGA2 antibody-directed liposomes

**Figure 4:**
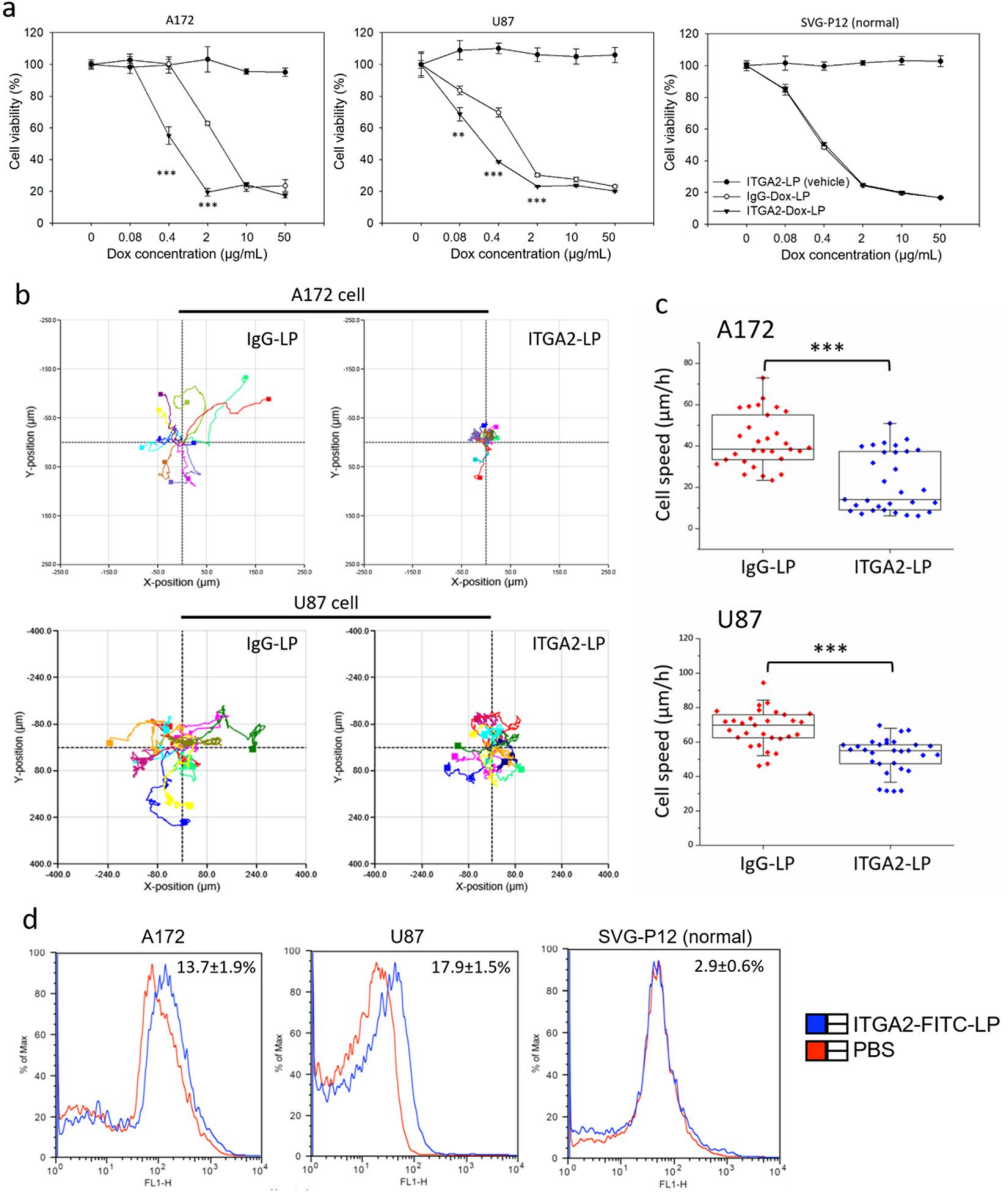
GBM-targeted therapy of ITGA2-Dox-LP. (**a**) Cytotoxicity of ITGA2-LP (vehicle), IgG-Dox-LP, and ITGA2-Dox-LP in A172, U87, and SVG-P12 (normal) cells. (**b**) Cell migration trajectories of A172 (upper panel) and U87 (lower panel) cells under the treatment of ITGA2-LP blockade. Significant reductions in cell migration were observed following ITGA2 blockade. IgG-LP was used as a control. (**c**) Quantified cell migration speed of A172 and U87 cells with or without ITGA2-LP blockade. (**d**) ITGA2-FITC-LPs transmitted across an *in vitro* BBTB (A172 and U87 cells) were quantified using flow cytometry analysis. Normal SVG-P12 cells were used as a control.

Toxicity of ITGA2-Dox-LP was evaluated by measuring its half maximal inhibitory concentration (IC_50_) in human GBM cells. In dose-dependent cytotoxicity studies (Fig. 4a), ITGA2-Dox-LP exhibited significantly increased toxicity than non-specific IgG-Dox-LP in A172 and U87 cells but not in the normal SVG-P12 cells due to their lack of ITGA2 expression. Empty liposome vehicles (ITGA2-LP) did not exhibit any toxic effects in all studied cell lines, suggesting the ITGA2 antibody conjugated immunoliposome itself is not cytotoxic. The IC_50_s of ITGA2-Dox-LP and IgG-Dox-LP were 0.40 and 2.02 µg/mL for A172 cells, 0.12 and 0.51 µg/mL for U87 cells, 0.28 and 0.26 µg/mL for SVG-P12 (normal) cells, respectively. The IC_50_ of ITGA2-Dox-LP for human GBM cells is similar to that of free doxorubicin (Table S1). However, it is critical to note that the cytotoxicity of free doxorubicin is non-specific and kills any cells contacted (both GBM and normal, indiscriminately). These data indicate that ITGA2 antibody-directed liposomes are over 4-times more effective in ablating GBM cells compared with non-specific IgG-Dox-LP, without affecting normal glial cells, suggesting that ITGA2-Dox-LP may be more selective and effective in treating GBM cells than the conventional non-targeting nanomedicines.

We also evaluated the therapeutic benefits of ITGA2 blockade from ITGA-Dox-LP. Empty ITGA2-LP or IgG-LP (without doxorubicin) were used to assess the impacts of ITGA2 blockade on GBM cell migration without the interference from cytotoxic doxorubicin. We used a quantitative phase imaging (QPI) assay^27,28^ to assess GBM cell migratory behaviors under the treatment of ITGA2-LP at an established antibody dose of 2 µg/mL^29^. As shown in Fig. 4b, the motility of A172 cells with ITGA2-LP or IgG-LP treatment were recorded as time-lapse rose plots, where IgG-LP-treated A172 cells showed a more dispersed pattern than ITGA2-LP-treated cells. This is due to the fact that these IgG-LP-treated GBM cells exhibit a 1.3–1.9 fold higher motility speed than ITGA2 antibody-blocked GBM cells (Fig. 4c), leading to a 24–47% increased migration distance. We further investigated the inhibitory effect of ITGA2-LP on human GBM cell migration activity at different incubation times (24 h and 48 h). As shown in Fig. S4, the inhibitory effect of ITGA2-LPs on A172 cell migration is potent and persistent, mediating approximately 42–47% cell migration reduction at both 24 h and 48 h. This suggests that ITGA2 antibodies maintain their neutralization activity after covalent conjugation on the surface of liposomes. These studies suggest that our engineered ITGA2-Dox-LPs may cooperatively inhibit GBM cell migration through the ITGA2 antibody and destroy these GBM cells via enhanced intracellular delivery of doxorubicin, which may lead to synergistic therapeutic effects in GBM therapy.

### Transmission across blood-brain tumor barrier (BBTB)

During *in vivo* GBM therapy, ITGA2-Dox-LPs must cross the BBTB before reaching GBM tumor region. We therefore evaluated the extravasation capability of ITGA2 antibody-directed liposomes in an established *in vitro* BBTB model^30,31^. It has been known that human GBM cells secrete a variety of pro-angiogenic factors (e.g., vascular endothelial growth factor, VEGF)^32^, which may disrupt the integrity of human brain microvascular endothelial cell (HBMVEC) barriers, leading to an increased transendothelial delivery of ITGA2-Dox-LP. As shown in Fig. 4d, when we co-cultured a pre-established HBMVEC barrier with GBM cells (A172 and U87 cells), we observed that a significant amount of ITGA2-FITC-LPs transmitted across the BBTB and were taken up by GBM cells. In comparison, there was no increased cell uptake of ITGA2-FITC-LP in SVG-P12 cells in a normal BBB model because normal SVG-P12 cells do not secrete pro-angiogenic factors and maintain high integrity of normal BBB^30,31^. These studies indicate that ITGA2-FITC-LPs may selectively breach the BBTB but not normal BBB due to the GBM-induced angiogenesis effects.

## Discussion

The ultimate goal of this study is to develop clinically safe, specific, and effective antibody-directed nanomedicines for GBM. One immediate and significant challenge impeding pursuit of this goal has been the lack of effective GBM-specific targets. Several GBM nanomedicines have been previously reported to utilize general cancer antigens such as CD44, EGFR, and transferrin receptor (TFRC)^14,33–36^. Unfortunately, a critical limiting problem is that these antigens (CD44, EGFR, and TFRC) are also expressed on normal brain tissue at high levels^37–39^, and consequently are non-specific and cannot differentiate between GBM and normal brain cells. Furthermore, though many cancer antigens have been investigated in the hope of finding a GBM-specific target, to date, there is a lack of systematic and quantitative analysis of the overexpression levels of these targets on GBM cells. As such, an optimal nanotherapeutic target for GBM has remained unidentified.

In this study, we have systematically evaluated a panel of 70 cancer-related antigens in GBM cells and have identified ITGA2 as a promising new target for GBM. ITGA2 is a heterodimeric transmembrane glycoprotein in the family of integrin alpha subunits, and it frequently forms heterodimer α2β1 with integrin beta 1 (ITGB1), with the two functioning together as collagen and laminin receptors to mediate cell adhesion to extracellular matrix (ECM)^40^. In normal tissues, ITGA2 has been found to be primarily expressed in skin, lung, salivary glands as well as activated endothelial cells but is absent on quiescent endothelial cells^19,41^. The expression of ITGA2 was found to be upregulated in several types of tumors (e.g., gastric cancer, prostate cancer, and non-small cell lung cancer) and it is closely associated with tumor invasion^19,41–43^. Recently, it has been reported that ITGA2 is upregulated in 375 human gastric tumor tissues and ITGA2 antibodies mediate potent cell apoptosis in human gastric cancer AGS cells through RhoA-P38 signaling pathways^19^. They also found that ITGA2 antibodies inhibit AGS cell migration and impede its actin organization. To date, the role of ITGA2 and its heterodimer α2β1 in malignant brain tumors is still not well understood. ITGA2 has not yet been identified as a GBM-specific target until this current study.

In contrast to previous potential GBM markers, ITGA2 differs by its notable tumor-specific expression and its high expression across multiple cell lines and samples. ITGA2 is significantly upregulated in both human GBM tumor tissues and cell lines and is uniformly minimally expressed in normal tissue and cells. Functionally, we found that antibody blockade of ITGA2 potently inhibits GBM cell migration. The relevance of these *in vitro* findings are reflected in the outcome of patients when clinical data are reviewed. By analyzing the R2: Genomics Analysis and Visualization Platform database, we found that ITGA2 expression is significantly upregulated in human GBM tumor tissues with high ITGA2 expression being closely associated with decreased GBM patient survival, suggesting that ITGA2 may have an important role in the development and progression of GBM. Our data suggest that ITGA2 may have utility as a clinically relevant therapeutic target. To explore the application of ITGA2 in GBM-targeted nanomedicines, we constructed ITGA2 antibody-directed, doxorubicin-encapsulating liposomes (ITGA2-Dox-LPs). Of note, the ITGA2 antibody conjugated on the liposome surface is not only a targeting ligand, but also has a therapeutic function itself: antibody blockade of ITGA2 on GBM cells potently inhibits GBM cell migration. Taken together, our novel ITGA2-Dox-LP is designed to synergistically inhibit both GBM cell proliferation and cell migration during GBM-targeted therapy. Our experiments demonstrated that these nanomedicine constructs were highly successful in selectively binding and ablating human GBM cells. Importantly, ITGA2-Dox-LPs also effectively breach an *in vitro* BBTB via GBM-induced angiogenesis effects^44,45^.

This novel precision nanomedicine has the potential to load a variety of therapeutic agents (e.g., chemodrugs and siRNAs) into the nanoliposomal drug delivery system and selectively deliver these payloads into the sites of diseases while limiting drug accumulation and exposure to normal organs and tissues. This may significantly reduce the treatment’s adverse effects and improve GBM therapeutic efficacy. The combination of a novel, highly tumor-specific target coupled with a precise delivery mechanism overcomes two of the major challenges limiting advances in GBM therapy. These proof-of-principle studies support the conclusion that ITGA2 may be used as a novel GBM target to develop more effective GBM-targeted nanomedicines.

## Conclusion

For the first time, we have identified and characterized ITGA2 as a novel molecular target for GBM that is robustly expressed in multiple representative GBM cell lines while being absent in normal glial cells. By analyzing R2: Genomics Analysis and Visualization Platform database, we found that ITGA2 is significantly upregulated in human GBM tumor tissues and high ITGA2 expression has a negative impact on GBM patient survival, suggesting ITGA2 as a promising therapeutic target for GBM. We have developed an ITGA2 antibody-directed, doxorubicin encapsulating liposome (ITGA2-Dox-LP) as a novel GBM-targeted nanomedicine that selectively binds and kills GBM cells *in vitro*. We also demonstrated that these GBM-targeted ITGA2-Dox-LP could effectively breach an *in vitro* BBTB via GBM induced angiogenesis effects^44,45^, but not a normal intact *in vitro* BBB. These findings may have significant clinical potential for GBM therapy and diagnosis and support further research into the use of ITGA2 as a therapeutic target for GBM.

## Materials and Methods

### Materials

Dulbecco’s phosphate buffered saline (PBS), 4′,6-diamidino-2-phenylindole (DAPI), 0.25% trypsin/2.6 mM ethylenediaminetetraacetic acid (EDTA) solution, Mouse anti-human ITGA2 antibody (clone#16B4), and Gibco® Dulbecco’s Modified Eagle Medium (DMEM) were purchased from Invitrogen (Carlsbad, CA, USA). 1-Ethyl-3-(3-dimethylaminopropyl) carbodiimide hydrochloride (EDC), N-hydroxysuccinimide (NHS), bovine serum albumin (BSA), anhydrous dimethyl sulfoxide (DMSO), doxorubicin (Dox), and fluorescein isothiocyanate–dextran (FITC-dextran, MW 10 kD) were purchased from Sigma-Aldrich (St. Louis, MO, USA). Corning® Transwell® polycarbonate membrane cell culture inserts, Lab-Tek II Chamber Slide System, formaldehyde, chloroform, anhydrous ethanol (EtOH), Slide-A-Lyzer dialysis cassette (MWCO 20 KD), and Diff-Quik Stain Set were purchased from Thermo Fisher Scientific (Pittsburgh, PA, USA). Mouse immunoglobulin G (IgG) isotype was purchased from R&D Systems (Minneapolis, MN, USA). Phycoerythrin (PE)-conjugated mouse anti-human antibodies against 70 cancer target candidates, and PE-conjugated mouse IgG isotypes were purchased from BioLegend (San Diego, CA, USA). 1,2-dioleoyl-sn-glycero-3-phosphocholine (DOPC) and 1,2-distearoyl-sn-glycero-3-phosphoethanolamine-N-[carboxy(polyethylene glycol)-2000] (DSPE-PEG-COOH) were purchased from Avanti Polar Lipids (Alabaster, AL, USA). Quantum Simply Cellular microbeads were purchased from Bangs Laboratory (Fishers, IN, USA). FLOAT-A-LYZER G2 dialysis tubing (MWCO 1,000 kDa) was purchased from Spectrum Laboratories (Rancho Dominguez, CA, USA). The Dojindo cell counting kit CCK-8 was purchased from Dojindo Molecular Technologies (Rockville, MD, USA).

### Cell culture

Human GBM cell lines (A172, U87, U118) and normal human glial cells (SVG-P12) were obtained from the American Type Culture Collection (ATCC, Manasses, VA, USA), while human brain microvascular endothelial cells (HBMVEC) were obtained from Cell Systems Inc. (Seattle, WA, USA). The A172, U87, U118, and SVG-P12 cell lines were cultured in Dulbecco’s Modified Eagle’s Medium (DMEM) with 10% Fetal Bovine Serum and a 1% concentration of Penicillin Streptomycin (Penstrep). The HBMVEC cells were cultured in Lonza EGM-2MV medium. All of the cell lines were maintained at 37 °C in a humidified incubator with 5% CO_2_.

### Flow cytometry measurements

The cell surface expression of 70 cancer-related antigens was evaluated using a BD FACSCalibur Flow Cytometer (BD Biosciences, San Jose, CA, USA) as previously described^13,46^. Surface antigen density on target cell surfaces was quantified using Quantum Simply Cellular microbeads as a reference and was conducted according to the protocol provided by the manufacturer. Briefly, 10^6^ GBM or SVG-P12 cells were harvested and washed twice with PBS. Following this rinsing, the cells were blocked in 1% bovine serum albumin (BSA) in PBS for 30 minutes in an ice bath. Post-blocking, cells were incubated with PE-conjugated antibodies for 1 h at room temperature. Following the incubation, the cells were rinsed with 1% BSA in PBS three individual times, resuspended in new PBS, and analyzed via flow cytometry.

### Immunofluorescent staining

200,000 GBM or SVG-P12 cells were seeded in a Lab-Tek II Chamber Slide System with 2 ml of full serum media overnight under standard culture conditions. Following aspiration of the media, cells were washed twice with PBS and fixed with 4% formaldehyde in PBS at room temperature for 10 minutes. Following the fixation, the fixed cells were washed twice with PBS and blocked with 1% BSA in PBS for 30 minutes in an ice bucket. Post-blocking, cell samples were stained with PE-conjugated ITGA2 antibody or PE-conjugated IgG for 1 hour and rinsed with PBS. To stain the cell nuclei, DAPI was utilized. Immunofluorescent stained samples were allowed to dry overnight in a dark room, and were then examined using a Leica TCS SP5 confocal fluorescent microscope (Leica Systems, Buffalo Grove, IL, USA). The digital staining images were obtained using Axiovision digital image processing software.

### Genomic analysis of ITGA2 in human GBM

The ITGA2 mRNA expression of human GBM tumors and normal brain tissues were analyzed using the R2: Genomics Analysis and Visualization Platform database (https://hgserver1.amc.nl/, Datasheet: Mixed Pediatric Brain (Normal-Tumor)-Donson-130-MAS5.0-u133p2). Gene expression data were generated from tumor and normal brain human samples using Affymetrix HG-U133plus2 chips (Platform GPL570)^17,18^. The clinical association of ITGA2 mRNA expression on GBM patient survival was analyzed using the same database (https://hgserver1.amc.nl/). Kaplan–Meier curves were generated using the following datasheet (Tumor Glioblastoma-TCGA-540-MAS5.0-u133a, a cohort of 540 patients). The detailed information of the microarray and RNA-Seq experiments can be found at the TCGA Data Portal (https://tcga-data.nci.nih.gov/tcga/). All genomic datasets used in our studies are publically available in the R2: Genomics Analysis and Visualization Platform database (https://hgserver1.amc.nl/).

### Cell proliferation assays

GBM and SVG-P12 cells were seeded in 96 well plates at a density of 3,000 cells per well and left to adhere overnight in the incubator with 5% CO_2_ at standard conditions. Following this incubation, the cells were treated with 2 µg/ml of ITGA2 or IgG antibodies in DMEM with 10% FBS and 1% Penstrep. Cell viability was determined at time intervals of 1, 3, and 5 days utilizing a Dojindo cell counting kit CCK-8 according to the protocol provided by the manufacturer.

To evaluate the cytotoxicity of GBM-targeted immunoliposomes, cell treatment groups were assigned and treated with (1) ITGA2 antibody-conjugated liposomes (vehicles), (2) non-specific IgG-conjugated, doxorubicin-encapsulating liposomes (IgG-Dox-LP), (3) ITGA2 antibody-conjugated, doxorubicin-encapsulating liposomes (ITGA2-Dox-LP) at the following equivalent doxorubicin doses 0.08, 0.4, 2, 10, 50 µg/ml for 6 hours. Cells were rinsed twice with PBS and grown for 48 hours before the Dojindo assay was performed.

### Cell migration assays

10^6^ GBM and SVG-P12 cells were pre-treated with free IgG and ITGA2 antibody, as well as antibody conjugated liposomes for 24 h at an antibody dose of 2 µg/mL. They were then seeded onto COSTAR transwell inserts with permeable support polycarbonate membrane with an 8 um pore size in a 24 well plate at a cell density of 10,000 cells per well with serum-free DMEM media. DMEM with 10% FBS was added to the lower wells as chemoattractants. The cells were allowed to migrate for 20 hours, at which point the inserts were stained with Diff-Quik Stain Set. The cells that did not migrate through the insert membrane were removed using cotton swabs, at which point four fields per insert were counted for each sample.

### Synthesis and characterization of ITGA2 antibody-directed liposomes

ITGA2 antibody-directed liposomes were prepared using an established nanoporous membrane extrusion method as previously described^24^. Briefly, a lipid mixture of DOPC:DSPE-PEG-COOH (95:5, mol:mol, 50 µmol in total) was dissolved in chloroform and dried under a dry nitrogen stream. The lipid film that resulted was solubized in 1 mL DMSO:EtOH (7:3). The solubized lipids were injected into 9 ml of 240 mM ammonium sulfate while being constantly and rigorously agitated to yield a 5 mM lipid solution. Following 5 freeze-thaw cycles, the lipid solution was extruded using a Lipex Extruder utilizing a 100 nm polycarbonate nanoporous membrane. Following the extrusion, the liposome solution was placed in dialysis in PBS using a Slide-A-Lyzer dialysis cassette (MWCO 20 kDa) and dialyzed overnight at room temperature. Doxorubicin was added to the liposome solution to reach a final concentration of 1 mg/ml and the combined mixture was incubated for 6 hours to allow for active loading of the doxorubicin. The resulting doxorubicin-encapsulating liposome solution was then dialyzed in PBS using the same Slide-A-Lyzer Dialysis cassette overnight at room temperature.

ITGA2 antibodies were covalently conjugated to the surface of the liposomes via the DSPE-PEG-COOH anchor. NHS (3 mg) and EDC (2 mg) were added to 5 µmol lipids of liposome solution and incubated for 10 min, then ITGA2 antibodies and IgG antibodies were added to the EDC-modified liposomes at a molar ratio of 1:1000 antibody:phospholipid and incubated 2 h at room temperature. Unreacted antibodies were removed by utilizing a FLOAT-A-LYZER G2 dialysis tubing unit (MWCO 1,000 kDa). For liposome binding experimentation, non-cytotoxic FITC-dextran encapsulating liposomes were prepared and tested in lieu of the cytotoxic doxorubicin-encapsulated liposomes. The preparation process was identical with the exception being that the 1 ml lipid solution was mixed with a 9 ml FITC-dextran solution (1 mg/ml).

The density of ITGA2 antibodies conjugated on the surface of the liposomes was quantified using a microbead assay as previously described^21,22^. 2 µm borosilicate beads were encapsulated with DOPC:DSPE-PEG-COOH (95:5, mol:mol) liposomes by rigorously agitating small unilamellar liposomes with the microbeads in PBS for 6 h. Then, the microbeads rinsed three times in PBS with suspension-spin cycles to separate free liposomes. Conjugation of the PE-IgG and PE-ITGA2 antibodies to microbead encapsulating liposomes utilized EDC/NHS chemistry. Surface density of ITGA-2 antibody conjugated to each microbead was ascertained with reference to Quantum Simply Cellular microbeads, which have defined numbers of antibody sites per bead. Liposome size and zeta potential were measured via dynamic light scattering on a ZETA-PALS analyzer (Brookhaven Instruments, Holtsville, New York).

### Quantitative phase imaging analysis

GBM cell migratory behaviors with ITGA2 antibody or IgG-conjugated liposome treatment was evaluated using an established quantitative phase imaging method as previously described^27,28,47^. Briefly, GBM cells were cultured in 6-well plates with a density of 50,000 cells per well. After allowing for attachment overnight, the six well plate was placed on a motorized stage of HoloMonitor® M4 (Phase Holographic Imaging Phi AB, Lund, Sweden) with a 20× objective lens. The system was kept in a humidified incubator with 5% CO_2_. HstudioM4 software was used to record phase images of the sample within the field of integration (0.5 mm^2^). For each well, 4 locations on the plate were selected to scan every 5 minutes to acquire continuous phase images for a period of 48 h. Cell morphology, migration, and proliferation characteristics were also documented and recorded for statistical analysis with ITGA2 antibody or IgG-conjugated liposome treatments (at equivalent antibody concentration of 2 µg/mL).

### *In Vitro* Blood-Brain tumor barrier assay

20,000 HBMVECs were seeded on attachment factor-coated transwell inserts with 3 μm pores and incubated for 48 h as previously reported^30,31,44^. 20,000 GBM or SVG-P12 cells were separately seeded in a well of 24-well plate for 24 h. The HBMVEC-coated transwell insert was then co-cultured with GBM or SVG-P12 cells in 24-well plate for 24 h to induce vascular permeability. 100 μL of ITGA2-FITC-LP in DMEM (0.5 mM lipids) and 600 μL of DMEM were separately added to the top and bottom sides of the HBMVEC-coated transwell inserts and further incubated for 4 h. The GBM and SVG-P12 cells in the bottom chamber were collected and ITGA2-FITC-LP translocated through the endothelial barrier was quantified by measuring their fluorescence using flow cytometry analysis^30,31,44^.

### Statistical analysis

All experimental data were collected in triplicate unless otherwise noted and are presented as mean +/− standard deviation. Statistical comparison by analysis of the variance was performed at a standard significance level of P < 0.05 utilizing the Student’s T-test.

## Supplementary information

Supplementary information
