# Supplementary material for: ITGA2 as a potential nanotherapeutic target for glioblastoma
Source: Sci Rep. 2019 Apr 17;9:6195. doi: 10.1038/s41598-019-42643-7 (PMC6470144; doi:10.1038/s41598-019-42643-7)

**Supplementary Information**

**ITGA2 as a potential nanotherapeutic target for glioblastoma**

Peng Guo, Alexander Moses-Gardner, Jing Huang, Edward R. Smith, and Marsha A. Moses

**Figure S1.** The effect of ITGA2 antibody blockade on human A172 cell proliferation at different antibody concentrations: (a) 0.4 µg/mL, (b) 2 µg/mL, and (c) 10 µg/mL. IgG was used as the control.
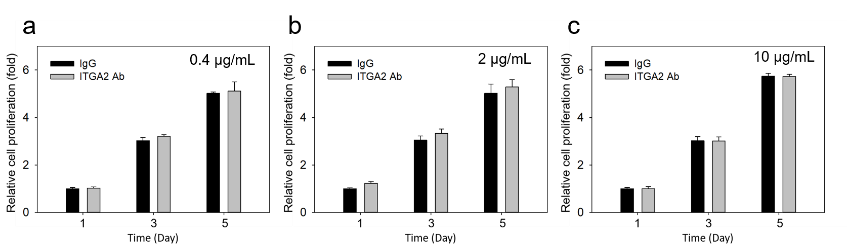


**.**

**Figure S2.** ITGA2 antibody blockade inhibits human A172 cell migration at different antibody concentrations: 0.4 µg/mL, 2 µg/mL, and 10 µg/mL. Representative microscope images (a) and quantitative analyses (b) of migrated A172 cells under ITGA2 antibody blockade treatment in a transwell migration assay. IgG was used as a control. *** P<0.001, NS not significant.


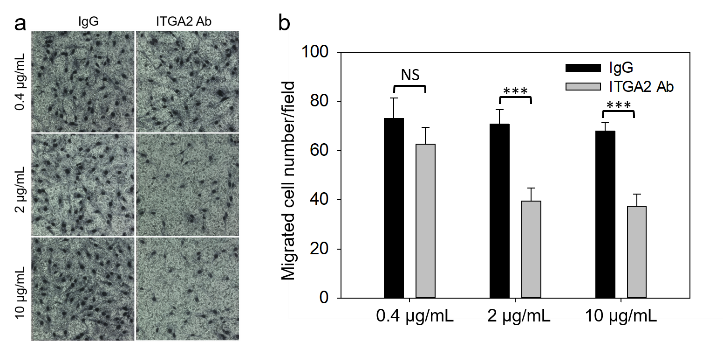


**Figure S3.** Representative TEM images of IgG-Dox-LP and ITGA2-Dox-LP. The scale bars represent 400 nm.


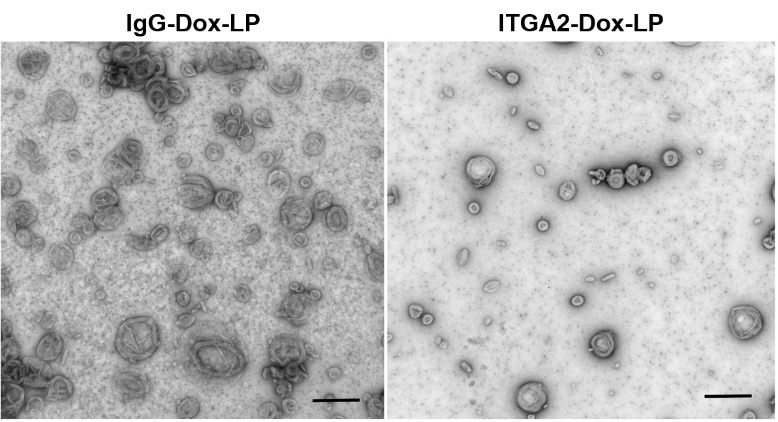


**Figure S4.** QPI analyses of human GBM cell migration activity at different liposome incubation times (24h and 48h). (a) Cell migration trajectories of A172 cells treated with IgG-LP or ITGA2-LP for 24h and 48h. (b) Quantified cell migration speed of A172 cells treated with IgG-LP or ITGA2-LP for 24h and 48h, demonstrating significantly decreased migration of glioma cells following ITGA2-LP treatment.

**
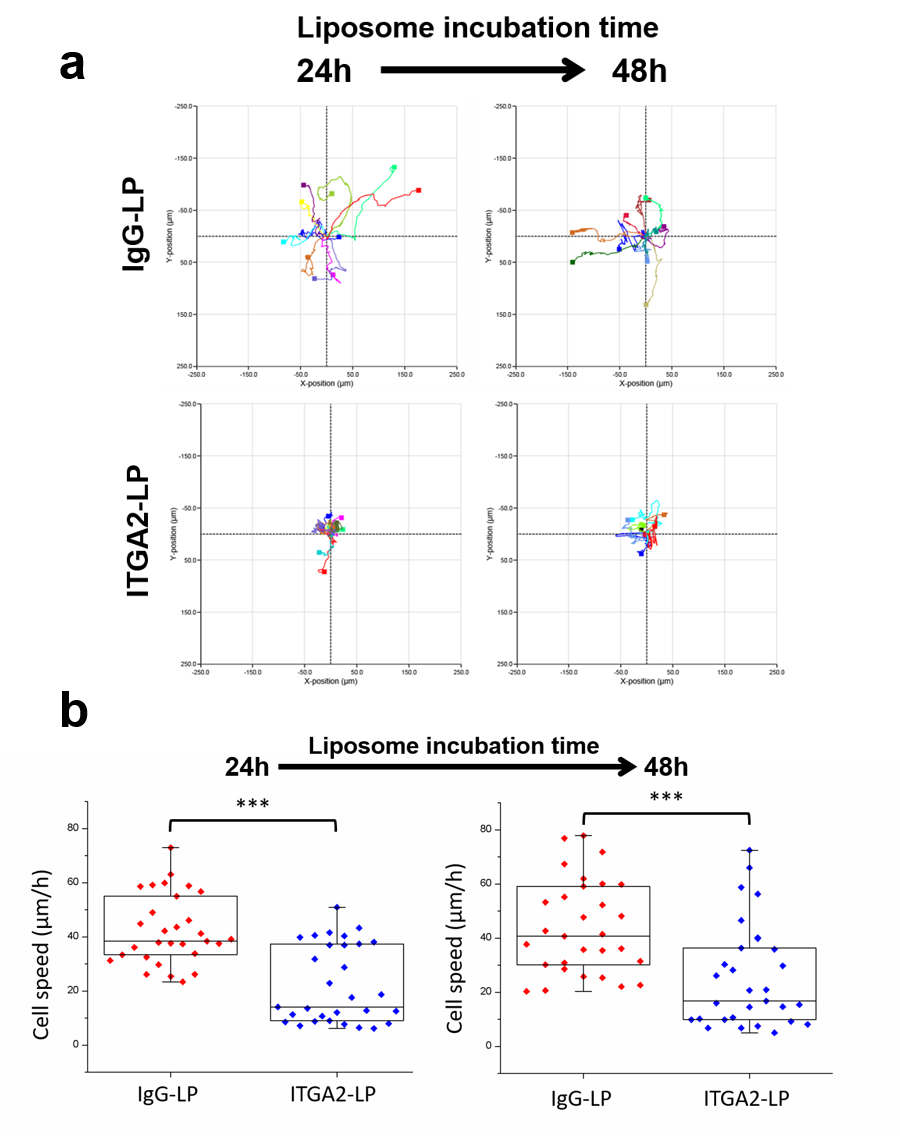
**

**Table S1.** IC_50_ values of free Doxorubicin, IgG-Dox-LP and ITGA2-Dox-LP on human GBM cells.


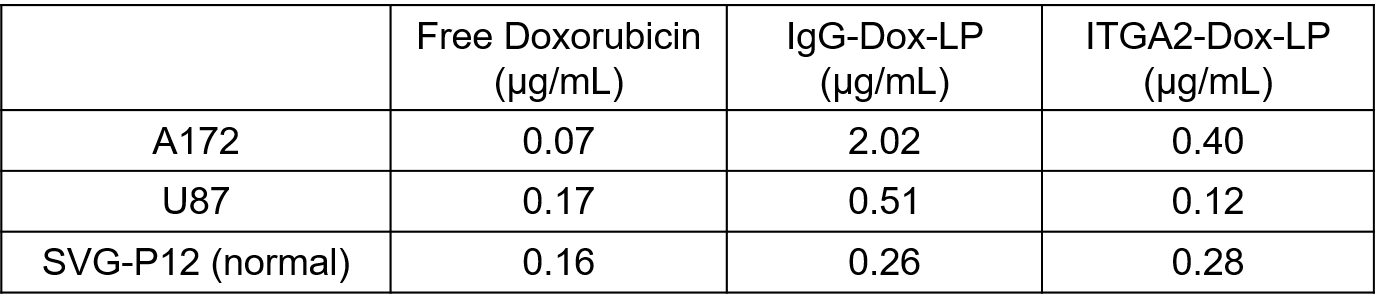

Supplement: Supplementary file 1 — Supplementary information [file 41598_2019_42643_MOESM1_ESM.docx]
